# The association of high-sensitivity C-reactive protein with future weight gain in adults

**DOI:** 10.1038/s41366-022-01101-7

**Published:** 2022-03-08

**Authors:** Riina Santa-Paavola, Susanna Lehtinen-Jacks, Tuija Jääskeläinen, Satu Männistö, Annamari Lundqvist

**Affiliations:** 1 Faculty of Medicine and Health Technology, Tampere University, Tampere, Finland; 2 School of Health Sciences, Faculty of Social Sciences, Tampere University, Tampere, Finland; 3 Division of Public Health Sciences, School of Health, Care and Social Welfare, Mälardalen University, Västerås, Sweden; 4 Finnish Institute for Health and Welfare, Helsinki, Finland

**Keywords:** Obesity, Obesity, Epidemiology, Weight management

## Abstract

**Background:**

Obesity is associated with low-grade systemic inflammation, and it has been suggested that increased inflammation markers could predict future weight gain. Our aim was to investigate the associations of high-sensitivity C-reactive protein (hs-CRP) concentration with changes in weight and waist circumference in adults during 11 years of follow-up.

**Methods:**

We used data from the Health 2000 and Health 2011 surveys consisting of a population-based sample of Finnish adults. We included those 3143 participants, aged 30–75 years at baseline, whose baseline hs-CRP was measured, and who had information on measured weight and height at both time points. Associations between baseline hs-CRP and changes in weight and waist circumference were analyzed using multinomial logistic regression, adjusted for sociodemographic factors (age, sex, marital status, and educational status), lifestyle factors (smoking, alcohol consumption, leisure-time physical activity, sitting time, sleeping time, and psychological distress), and baseline values of BMI and waist circumference.

**Results:**

Hs-CRP was not associated with weight gain (≥5%) when adjusted for potential confounders (OR 0.99, 95% CI 0.96–1.01), compared to stable weight (change <±5%). Higher baseline hs-CRP was associated with decrease in weight (≤−5%) in the unadjusted (OR 1.03, 1.01–1.05), but not in the adjusted (OR 1.01, 0.99–1.03) model. No association was observed between hs-CRP and waist circumference.

**Conclusions:**

Hs-CRP was not associated with future changes in weight or waist circumference in adults. These findings suggest that hs-CRP concentration does not predict future weight gain.

## Introduction

Overweight and obesity are a growing global public health problem. According to the WHO´s estimates, 39% of adults aged 18 years and older had overweight (BMI 25.0–29.9 kg/m^2^) and 13% had obesity (BMI ≥ 30 kg/m^2^) in the year 2016 [1]. The problem is more common in the higher-income countries, but it is continuously increasing in other parts of the world [2, 3]. In Finland, every fourth adult had obesity and almost half of the adults had central obesity in the year 2017 [4].

Obesity is a risk factor for many chronic diseases, such as cardiovascular diseases, type 2 diabetes, musculoskeletal disorders, chronic kidney disease and some cancers, and it is associated with increased risk of premature mortality [5–8]. Obesity is also related to higher risk of mental health problems and lower health-related quality of life [9–11].

Major risk factors of obesity, such as genetics, high energy diet, physical inactivity and limited amount of sleep, are well known [12–15]. Furthermore, factors like low socioeconomic status, depression, and stress are associated with increased risk of obesity [16–18].

In cross-sectional studies, persons with obesity have shown to have increased concentrations of inflammation markers, such as high-sensitivity C-reactive protein (hs-CRP), compared to persons with normal weight [19–21]. It is hypothesized that increased amount of adipose tissue is the cause of the systemic inflammation [22, 23]. On the other hand, some longitudinal studies have suggested that elevated inflammation markers are associated with future weight gain [24, 25]. These results raise the question of whether low-grade systemic inflammation could be a risk factor for obesity, not only a consequence of it. Further, in some studies an association between higher levels of inflammation markers and weight gain has been seen particularly among those who had recently stopped smoking [25, 26].

The aim of the present study was to investigate the associations of hs-CRP concentration with future weight and waist circumference changes in adults during an 11-year follow-up period. We included in the statistical models a wide range of health-related and behavioral factors, such as smoking, leisure time physical activity, alcohol consumption, sleeping and psychological distress, which could confound the association between hs-CRP concentration and changes in weight and waist circumference.

## Materials and methods

This study is based on the Health 2000 and Health 2011 surveys conducted in Finland between 2000 and 2011 [27, 28]. For the Health 2000 survey, a sample representing the Finnish adult population living in mainland Finland was drawn from the Finnish Population Registry using a two-stage stratified sampling. The sample comprised 9922 persons aged 18 or over, of whom 8028 were at least 30 years old. The survey was carried out in 80 study areas all over the country and a total of 84% of the sample aged 30 or over participated in a health examination. All members of the Health 2000 sample who were not dead or, living abroad, and had not refused further studies, were invited to the Health 2011 survey (*n* = 8135) and 59% of them participated in a health examination.

**Fig. 1:**
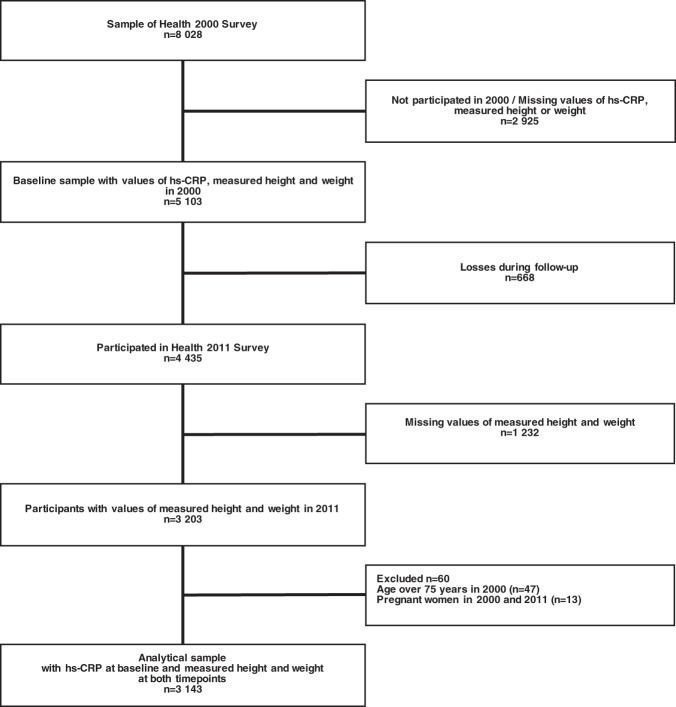
Participants‘ selection process and number of exclusions.

In the present study, we included those participants of the Health 2000/2011, who had taken part in both surveys, whose baseline hs-CRP concentration was measured in 2000, and who had measured weight and height information at both time points (Fig. 1). We excluded participants who were older than 75 years in 2000, or pregnant in 2000 or 2011. The final data consisted of 3143 participants. Among these persons, 3124 had waist circumference information available at both time points.

The main exposure in this study was hs-CRP. The concentration of hs-CRP was measured from blood samples taken at the baseline health examination in 2000. Serum samples were frozen to −70 °C, and hs-CRP was determined in 2003 by an immunoturbidimetric test in the Biochemistry Laboratory, THL. Details of the laboratory procedures have been presented in a previous study [29]. The analytical sensitivity of the test was 0.2 mg/l, while the data also included hs-CRP values, which were under 0.2 mg/l. In the statistical analyses, we assumed that these values are divided normally around 0.1 mg/l, and all the values under 0.2 mg/l in the data were replaced by the value of 0.1 mg/l.

The main outcome measures were change in weight (kg) and change in waist circumference (cm). Participants‘ weight, height, and waist circumference were measured by trained study nurses in the health examinations in 2000 and 2011. Height was measured without socks using a wall-mounted stadiometer and was recorded to an accuracy of 0.5 cm. Weight was measured using a bioimpedance device’s scale, or during a home-visit, using a digital scale. Weight was recorded to an accuracy of 0.1 kg. BMI was calculated as body weight divided by the squared value of body height (kg/m^2^). Waist circumference was measured in standing position, with the legs slightly apart and during light expiration, using a regular, flexible tailor’s measuring tape. The measurement was performed at the mid-point between the lowest rib bones and the highest point of iliac crest and was recorded to an accuracy of 0.5 cm.

Changes of weight and waist circumference were calculated as the difference in the respective values between the two time points, and the differences were presented as percentage of the baseline value. These variables were categorized into three categories for the statistical analyses, as follows: Weight change: weight loss (x ≤ −5%), stable weight (−5% < x < 5%) and weight gain (x ≥ 5%). Waist circumference: decrease (x ≤ −5%), stable (−5% < x < 5%) and increase (x ≥ 5%). For the descriptive analysis, BMI was categorized into five categories (underweight (<18.5 kg/m^2^), normal (18.5–24.9 kg/m^2^), overweight (25.0–29.9 kg/m^2^), obese (30.0–34.9 kg/m^2^), severely obese (≥35.0 kg/m^2^)) [30], and waist circumference into two categories (normal (men ≤ 100 cm, women ≤ 90 cm), central obesity (men > 100 cm, women > 90 cm)) [31].

The following factors were considered as potential confounders: sociodemographic factors (age, sex, marital status, educational status), lifestyle factors (smoking, alcohol consumption, leisure-time physical activity, sitting time, sleeping time, psychological distress). Age and sex were obtained from the Finnish population register, while the other information was collected through the Health 2000/Health 2011 interviews and questionnaires. Self-reported marital status was recategorized into two categories (married or cohabiting; never married, divorced or widowed) and educational status was used as a three-category variable (basic, intermediate, high). Participants were categorized into four categories by smoking status (daily, occasionally, ex-smoker, never smoker) and into three categories by the weekly alcohol intake (no use, moderate use (100% ethanol; women < 70 g, men < 140 g), risk use (women ≥ 70 g, men ≥ 140 g)). Leisure-time activity was recategorized into low (reading, watching TV or working in the household without much physical activity), moderate (walking, cycling, or other light exercise at least 4 h per week), and high (fitness training, intense training or sports competitions for at least 3 h a week). Time of sitting in hours per day was used as a continuous variable, because there is no consensus on a cut off point for an amount of sitting that is harmful to health. Sleeping time was categorized into three categories (short (<6 h), recommended (6–9 h), excessive (>9 h)) and the General Health Questionnaire (GHQ-12) score was categorized into two groups (<3 points, ≥3 points) describing psychological distress [32].

The data were described by mean values and standard deviations (continuous variables) and frequencies (categorical variables). Hs-CRP concentrations were presented as median and interquartile range, because of skewed distribution. The baseline characteristics of participants and non-participants (persons who had hs-CRP, weight and height measured in 2000 but did not have information of weight and height in 2011 available, persons who were more than 75 years old in 2000, and persons who were pregnant in 2000 or 2011 (*n* = 1960)) were compared by using Chi-square, ANOVA and Kruskal–Wallis tests. Changes in weight and waist circumference in men and women were presented as mean values and standard deviations, and as frequencies, and compared by *T*-test and Chi-square test, respectively. Associations of the main exposure (hs-CRP) and outcome variables (changes in weight and waist circumference) with covariates (potential confounders) were tested by using Pearson’s and Spearman’s correlation coefficient, Chi-square, ANOVA, and Kruskal–Wallis tests. The associations of hs-CRP with changes in weight and waist circumference were examined using two multinominal logistic regression analyses. In the first one, the dependent variable was the categorized weight change and in the other one, it was the categorized waist circumference change. The category of stable weight or waist circumference, respectively, was used as the reference group. In both multinominal regression analyses, four different models were applied; unadjusted, adjusted for age and sex, adjusted for all sociodemographic factors and lifestyle factors, and adjusted additionally for the baseline BMI or waist circumference, respectively. These multinominal regression analyses were also run separately for men and women.

Potential effect modification by smoking on the association between hs-CRP and weight change was investigated as follows. First, we added an interaction term between smoking and hs-CRP to the main multinomial regression model and assessed the statistical significance of the interaction term. Second, we created a new smoking variable, which takes into account the smoking status also at the follow-up. The smoking status was categorized as follows: smoker (smoker at both baseline and follow-up, *n* = 471), ex-smoker at baseline (*n* = 685), new quitter (smoker at baseline and ex-smoker at follow-up, *n* = 249), and never smoker (*n* = 1565). We then ran multinominal logistic regression analyses with the categorized weight change as the outcome variable in each smoking category separately.

In addition, we made the following additional and sensitivity analyses. To ensure that using hs-CRP as categorized vs. continuous variable did not affect the main results, multinominal regression analyses were re-ran with a categorical hs-CRP variable. Hs-CRP was categorized into quarters (Q1: < 0.25 mg/l, Q2: 0.25–0.64, Q3: 0.65–1.62, Q4: > 1.62) and the upper three quarters were compared to lowest one (Q1). We also divided participants into two groups according to the average (hs-CRP <1 mg/l vs. hs-CRP ≥1 mg/l) and high (hs-CRP ≤ 3 mg/l vs. hs-CRP > 3 mg/l) cardiovascular disease risk thresholds defined by the American Heart Association (AHA), and re-ran the multinominal logistic regression analyses using the respective lower category as the reference group [33]. Further, we ran linear regression analyses, where both the exposure (hs-CRP) and the outcome (change in weight (%) or change in waist circumference (%)) were modeled as continuous variables. Because the risk of diseases that could lead to weight loss increases with age, we also tested the interaction between age and hs-CRP in relation to weight change in two different ways: using age as a continuous variable, and using age as a binary variable based on median (≤48 years vs. >49 years).

A sensitivity analysis was performed to ensure that acute inflammation did not affect the main results. This was done by excluding from the main analysis all participants with a baseline hs-CRP ≥ 10 mg/l (*n* = 67), which is considered as an indicator of acute inflammation process. We also carried out a sensitivity analysis that excluded participants with cancer in active treatment, diagnosis of diabetes and treatment of CVD within a year (myocardial infarction, coronary artery disease, cardiac failure, or arterial hypertension) in 2000 or 2011 (*n* = 1000). Finally, we made two separate multinomial logistic regression models, to see whether (a) excluding leisure time physical activity and sitting time from the covariates, (b) adding fasting insulin value at baseline as a covariate, would affect the main results.

All analyses were conducted using the IBM SPSS Statistics 26 software and all the *P* values were compared to a significance level of 5%.

The Health 2000 Survey was approved by the Ethical Committee for Research in Epidemiology and Public Health, and the Health 2011 Survey by the Coordinating Ethics Committee at the Hospital District of Helsinki and Uusimaa (HUS). Written informed consent was obtained from all the participants.

## Results

At baseline, the mean age was 48 years and 54% of the participants were women (Table 1). In all, 60% of the participants had overweight (BMI ≥ 25 kg/m^2^) and 35% had central obesity (waist circumference in men >100 cm, in women >90 cm). A higher percentage of men had overweight than women (66% vs. 55%), while there was no difference in the prevalence of central obesity between men and women. The median hs-CRP concentration was 0.64 mg/l (IQR 1.38) among all participants, with no statistically significant difference between men and women (Table 2).

**Table 1:** Baseline characteristics of the participants and non-participants in the study.

	Participants *n* = 3143	Non-participants^a^ *n* = 1960	*p* value
Demographics			
Age (years)	48.2 (11.3)	57.7 (17.5)	<0.001
Sex (%)			0.537
Man	45.6	46.5	
Woman	54.4	53.5	
Marital status (%)			<0.001
Married or cohabiting	77.0	61.1	
Never-married, divorced or widowed	23.0	38.9	
Educational status (%)			<0.001
Basic	29.1	51.4	
Intermediate	35.6	28.4	
High	35.3	20.2	
Physical attributes			
hs-CRP concentration (mg/l)	0.64 (1.38)	0.96 (2.34)	<0.001
Weight (kg)	76.5 (15.1)	76.4 (16.0)	0.757
Height (cm)	169.3 (9.4)	166.7 (10.2)	<0.001
BMI (kg/m^2^)	26.6 (4.4)	27.4 (4.9)	<0.001
BMI category (%)			<0.001
Underweight (<18.5)	0.5	0.8	
Normal (18.5–24.9)	39.6	31.9	
Overweight (25.0–29.9)	39.5	42.1	
Obese (30.0–34.9)	15.9	18.5	
Severely obese (≥35.0)	4.5	6.6	
Waist Circumference (cm)	91.3 (12.9)	94.3 (13.5)	<0.001
Central obesity (men WC > 100, women WC > 90) (%)	34.8	45.0	<0.001
Lifestyle factors			
Smoking status (%)			<0.001
Daily	20.1	24.8	
Occasional	4.7	3.3	
Ex-smoker	21.9	21.8	
Never-smoker	53.3	50.1	
Leisure-time activity (%)			<0.001
Low	22.6	34.1	
Moderate	55.8	52.9	
High	21.6	13.0	
Alcohol consumption (%)			<0.001
No alcohol	24.9	41.1	
Moderate use (women < 20/day, men < 40 g/day)	66.8	50.1	
Risk use (women ≥ 20 g/day, men ≥ 40 g/day)	8.3	8.8	
Sleep, h/night (%)			<0.001
<6	2.9	4.7	
6–9	94.9	90.6	
>9	2.2	4.7	
Sitting time (h/day)	5.6 (3.2)	5.4 (3.0)	0.002
Psychological distress, GHQ ≥ 3 (%)	16.9	21.8	<0.001
Health			
Diabetes mellitus (diagnosed) (%)	3.1	8.4	<0.001
Cancer (in active treatment) (%)	1.8	3.3	0.001
CVD 1999–2001 (%)			
Myocardial infarction	0.2	0.9	<0.001
Coronary artery disease	0.6	1.0	0.069
Cardiac failure	0.2	1.3	<0.001
Arterial hypertension	3.9	3.0	0.091

Results of continous variables are presented as means (standard deviation), except hs-CRP, which is presented as median and interquartile range. Categorical variables are presented as percentage frequencies. The values of men and women are compared using *T*-test, Mann–Whitney test, and Chi-square. Missing value n ranges in participants from 0 to 166 and non-participants from 0 to 248 depending on variable.

^a^Persons, who have hs-CRP, weight and height measured in 2000, but do not have information of weight and height in 2011 available, or persons, who were over 75 years of age in 2000 or pregnant in 2000 or 2011.

**Table 2:** Baseline values of hs-CRP, weight, BMI and WC and changes of weight and WC during the 11-year-long follow-up.

	All (*n* = 3143)	Men (*n* = 1433)	Women (*n* = 1710)	*p* value^a^
Baseline				
hs-CRP (mg/l)	0.64 (1.4)	0.65 (1.2)	0.63 (1.6)	0.466
Weight (kg)	76.5 (15.1)	84.5 (13.3)	69.9 (13.2)	<0.001
BMI (kg/m^2^)	26.6 (4.4)	27.0 (3.9)	26.3 (4.9)	<0.001
WC (cm)	91.3 (12.9)	97.0 (10.9)	86.6 (12.6)	<0.001
Changes over follow-up				
∆ Weight (%)	2.6 (9.2)	1.4 (8.2)	3.5 (9.9)	<0.001
∆ Weight (%)				<0.001
≤−5	18.3	19.3	17.5	
−5 < x < 5	47.2	53.0	42.3	
≥5	34.5	27.7	40.2	
∆ WC (%)^b^	3.0 (8.4)	2.4 (7.2)	3.5 (9.2)	<0.001
∆ WC (%)^b^				<0.001
≤−5	15.1	13.3	16.7	
−5 < x < 5	48.6	55.0	43.3	
≥5	36.3	31.8	40.0	

Results of continous variables are presented as means (standard deviation), except hs-CRP, which is presented as median and interquartile range. Categorical variables are presented as percentage frequencies.

^a^Values of men and women are compared using *T*-test, Mann–Whitney test, and Chi-square.

^b^Missing values men *n* = 10 and women *n* = 9.

Compared to the participants, the non-participants were older and more often never married, divorced or widowed, and less educated, and had in general unhealthier lifestyles (Table 1). They also had higher hs-CRP, BMI, and prevalence of central obesity at baseline.

At baseline, hs-CRP concentration had a statistically significant correlation with BMI (*r* = 0.424, *p* < 0.001). Baseline hs-CRP values were higher in participants with overweight (Md 0.7 mg/l (IQR 1.3)), obesity (1.3 (2.2)) or severe obesity (2.7 (3.9)), compared to participants with normal weight (0.3 (0.7)). Hs-CRP concentration correlated also with waist circumference (*r* = 0.397, *p* < 0.001) and participants, who had central obesity (1.3 (IQR 2.2)) had higher hs-CRP compared to participants who did not (0.4 (1.0)). In addition, higher hs-CRP was associated with lower educational status and with leisure time inactivity.

Mean weight change over the 11 years follow-up was +2.6% (+1.8 kg) of the baseline weight and the respective change in waist circumference was +3.0% (+2.5 cm) (Table 2). Changes of weight and waist circumference were significantly higher in women compared to men. A higher proportion of women gained weight and increased waist circumference by 5% or more, while men were more likely to remain stable weight.

Table 3 shows odds ratios for the associations of baseline hs-CRP with changes in weight or in waist circumference, compared to stable weight or stable waist circumference, respectively. Hs-CRP was not associated with weight gain in the unadjusted model (OR 0.99, 95% CI 0.96–1.01), nor in the adjusted model (0.99, 0.96–1.01). Higher hs-CRP was associated with higher odds of losing weight in the unadjusted analysis (OR 1.03, 1.01–1.05). This association was slightly weaker after adjusting for sociodemographic and lifestyle factors (1.02, 1.00–1.04), and no longer significant after further adjusting for baseline BMI (OR 1.01, 0.99–1.03). Hs-CRP was not associated with increase (adjusted OR 1.01, 0.99–1.03) or decrease (adjusted OR 0.98, 0.95–1.01) in waist circumference. These results were similar in men and in women.

**Table 3:** Association (odds ratio, OR) of hs-CRP with weight and WC change using multinominal logistic regression, stable weight and stable WC (−5% < x < 5%) as the respective comparison group.

Change in weight	Weight loss ≥ 5%		Weight gain ≥ 5%	
Model	OR (95% CI)	*P* value	OR (95% CI)	*P* value
Unadjusted	1.03 (1.01–1.05)	0.008	0.99 (0.96–1.01)	0.248
Adjusted with age and sex	1.02 (1.00–1.04)	0.048	1.00 (0.98–1.02)	0.855
Adjusted with sociodemographic factors and lifestyle factors	1.02 (1.00–1.04)	0.110	0.99 (0.96–1.01)	0.364
Adjusted with sociodemographic factors, lifestyle factors, and baseline BMI	1.01 (0.99–1.03)	0.407	0.99 (0.96–1.01)	0.324
Change in WC	WC decrease ≥ 5%		WC increase ≥ 5%	
*Model*	OR (95% CI)	*P* value	OR (95% CI)	*P* value
Unadjusted	1.01 (0.98–1.03)	0.596	1.00 (0.98–1.02)	0.953
Adjusted with age and sex	1.00 (0.98–1.03)	0.975	1.01 (0.99–1.03)	0.467
Adjusted with sociodemographic factors and lifestyle factors	1.00 (0.97–1.03)	0.939	1.00 (0.98–1.02)	0.732
Adjusted with sociodemographic factors, lifestyle factors, and baseline BMI	0.98 (0.95–1.01)	0.253	1.01 (0.99–1.03)	0.437

Adjusted with sociodemographic factors and lifestyle factors includes age, sex, marital status, educational status, smoking status, leisure-time activity, alcohol consumption, sleep, sitting time, and psychological distress.

There was no statistically significant interaction between smoking at baseline and hs-CRP in relation to weight change during the follow-up period in the adjusted multinomial regression model (*p* = 0.740). There was neither significant association between hs-CRP and weight change in the adjusted models run separately among smokers, ex-smokers at baseline, new quitters, and never smokers.

The results received when using hs-CRP categorized into quarters were similar to the results with a continuous hs-CRP variable, with one exception: The highest hs-CRP quarter (Q4: > 1.62) was associated with higher odds of decrease in waist circumference; however, this association disappeared after the adjustments (Supplementary Table 1). Using sex-specific hs-CRP quartiles produced similar results. The results received when using hs-CRP dichotomized according to the AHA thresholds were similar to the results with a continuous hs-CRP variable, and the results received from the linear regression analyses using weight change (%) and waist circumference change (%) as continuous variables were consistent with the results from the multinomial logistic regression. There was no significant interaction between age and hs-CRP in relation to weight change during the follow-up period, whether using age as a continuous (*p* = 0.741) or a categorized variable (*p* = 0.415).

The exclusion of participants with hs-CRP ≥ 10 mg/l (*n* = 67) or the exclusion of participants with cancer in active treatment, diagnosis of diabetes and treatment of CVD within a year in both time points (*n* = 1000) did not affect the results of main analyses. Finally, excluding leisure time physical activity and sitting time from, or adding fasting insulin at baseline to the multinomial logistic regression model did not affect the results.

## Discussion

We studied changes in measured weight and waist circumference among more than 3000 Finnish adults, and across 11 years. Hs-CRP concentration at baseline was not associated with future weight gain. Participants, who lost at least 5% of their weight were more likely to have higher hs-CRP concentration, compared to those with stable weight. However, this association was explained by other factors, by higher baseline BMI in particular. Hs-CRP was not associated with change in waist circumference.

Several studies have shown that obesity leads to low-grade systemic inflammation [19–21]. Excess adipose tissue creates a proinflammatory condition, increasing the production of proinflammatory cytokines, which induces a chronic low-grade systemic metabolic inflammation, indicated by increased inflammation factor levels, such as hs-CRP [22, 23]. It has been suggested that this chronic low-grade systemic metabolic inflammation could also lead to future weight gain [24, 25].

However, previous epidemiological evidence on hs-CRP and weight gain is limited and contradictory. The few existing studies have reported similar and contradictory results to ours. The results of a Finnish population-based study on associations of several inflammation markers including hs-CRP with future weight gain (self-reported weight) during a 7-year follow-up among 25–75 years old adults (*n* = 3369) are consistent with ours, such that no associations between hs-CRP and obesity indicators were observed after adjusting for baseline BMI [34]. On the contrary, in a study from the US (*n* = 3254), higher baseline CRP concentration was associated with both weight loss and weight gain. In addition, higher baseline white blood cell count was associated with weight loss, and higher baseline fibrinogen and factor VIIIc values were associated with weight gain. However, the follow-up time of this study was only 3 years, and the participants were all at least 65 years old [35].

In two studies, the association between higher levels of inflammation markers and weight gain was seen particularly among those who had recently stopped smoking, as follows. In a German study with 25–75-year-old participants (*n* = 2792), higher baseline CRP concentration, fibrinogen and leukocytes were associated with weight gain during a 10-year follow-up. These associations were strongest among persons who quit smoking during the follow-up. No associations were observed between the inflammation markers and weight loss or changes in waist circumference in that study [25]. CRP was not included in a US study with a 3-year follow-up of 45–64-year-old individuals (*n* = 11,687), however, higher baseline levels of fibrinogen and leukocytes were associated with higher weight gain particularly among those who recently had quit smoking [26]. Our results do not confirm the findings from these two previous studies, but are in line with another Finnish study where smoking status did not affect the association between hs-CRP and weight change [34].

The present study has several strengths. First, the study population was large and we included only participants whose weight, height and waist circumference were measured by trained nurses, to avoid bias caused by underreporting of weight [36]. A clinically significant weight change was defined as at least 5% decrease or increase in weight, in accordance with several previous studies showing that weight loss of only 5% may bring beneficial changes in metabolism [37–39]. Further, change in waist circumference was used as another outcome to assess changes in central obesity. Central obesity is an independent health risk, because visceral fat gathered around the internal organs in the abdominal cavity is more metabolically active than fat in other parts of the body [40]. Also, we made several additional and sensitivity analyses to exclude that our methodological choices would explain the results.

Our study also has certain limitations. First, we only used one inflammation marker, hs-CRP, to assess low-grade inflammation. However, hs-CRP is widely used in inflammation diagnostics, and it has been shown that people with obesity have higher hs-CRP levels on average than persons with normal weight [19, 21, 41]. The very question in the present study was whether higher hs-CRP levels could predict future weight gain. Another limitation is that our data did not include information about whether a change in weight or waist circumference was intentional, nor about how much of the change occurred in fat vs. muscle tissue. The weight and waist circumference were measured also only twice, and we did not have information about the participants’ weight development between the two time points, nor earlier in their lives. Finally, the follow-up period was relatively long, 11 years, with no measurement points in between. It is possible that hs-CRP is associated with weight changes in shorter term, which could remain unobserved in the present study.

The original Health 2000 sample represents the Finnish adult population. The non-participant analysis showed that the participants were younger, higher educated and had healthier lifestyles than the non-participants. In addition, overweight and central obesity at the baseline were less common, and the hs-CRP concentration was lower among the participants. Therefore, persons included in the present analyses might be healthier than the general population, and it could be more difficult to observe an association between hs-CRP and changes in weight.

In conclusion, hs-CRP did not independently predict future weight gain or change in waist circumference during an 11-year follow-up among Finnish adults older than 30 years at the baseline.

## Supplementary information

Supplementary Table 1
